# Speech‐Language Pathology Students’ Knowledge & Attitudes about Dementia

**DOI:** 10.1002/alz70860_105267

**Published:** 2025-12-23

**Authors:** Larissa M Jordan

**Affiliations:** ^1^ University of Cincinnati, Joplin, MO, USA

## Abstract

**Background:**

Speech‐language pathologists (SLPs) provide assessment and intervention for communication and swallowing disorders that individuals with Alzheimer's disease and related dementias (ADRDs) experience. SLP students must have adequate knowledge and confidence in order to successfully work with individuals with dementia and their families.

**Method:**

Undergrad and graduate SLP students in both on campus and online cohorts will be asked to participate in an anonymous online survey. The survey will assess student's attitudes regarding ADRDs via the “Dementia Attitudes Scale” (DAS; O’Connor & McFadden, 2010) and the “Alzheimer's Disease Knowledge Scale” (ADKS; Carpenter et al., 2009). The DAS is a 20‐question inventory that evaluates dementia knowledge and social comfort via a 7‐point Likert scale (O’Connor & McFadden, 2010). The ADKS is a 30‐item true/false questionnaire which examines topics such as risk factors, assessment, symptoms, caregiving, and management (Carpenter et al., 2009).

**Result:**

This study is to be completed during the spring and summer of 2025. Findings regarding students’ attitudes and understanding of ADRDs will impact future changes to coursework by pinpointing areas for improvement.

**Conclusion:**

As the number of individuals with ADRDs continues to increase, the urgency to ensure SLP students are adequately trained to work with these populations continues to grow. We must prepare future SLPs and all future healthcare professionals with the knowledge and confidence to provide competent assessment and intervention for individuals with ADRDs.

Carpenter, B. D., Balsis, S., Otilingam, P. G., Hanson, P. K., & Gatz, M. (2009). The Alzheimer's Disease Knowledge Scale: development and psychometric properties. *The Gerontologist*, *49*(2), 236–247. https://doi.org/10.1093/geront/gnp023

O'Connor, M. L., & McFadden, S. H. (2010). Development and psychometric validation of the Dementia Attitudes Scale. *International Journal of Alzheimer's Disease, 2010*, Article 454218. https://doi.org/10.4061/2010/454218

